# Pregnancy in Pulmonary Arterial Hypertension: A Multidisciplinary Approach

**DOI:** 10.3390/jcdd9060196

**Published:** 2022-06-18

**Authors:** Yasmeen Daraz, Sandhya Murthy, Diana Wolfe

**Affiliations:** 1 Department of Medicine, Montefiore Medical Center, Albert Einstein College of Medicine, Bronx, NY 10467, USA; 2 Division of Cardiology, Montefiore Medical Center, Albert Einstein College of Medicine, Bronx, NY 10467, USA; smurthy@montefiore.org; 3 Division of Maternal Fetal Medicine, Department of Obstetrics and Gynecology, Montefiore Medical Center, Albert Einstein College of Medicine, Bronx, NY 10467, USA; dwolfe@montefiore.org

**Keywords:** pregnancy, pulmonary arterial hypertension, interdisciplinary, right ventricular failure, high-risk pregnancy

## Abstract

Pulmonary arterial hypertension (PAH), a female predominant disease, carries a high maternal and fetal mortality in pregnancy despite improved insight and the development of novel therapies. The high risk is attributed to the adaptive changes that take place to promote healthy fetal development during pregnancy, which can adversely affect the already compromised right ventricle in patients with PAH. While in the prior era emphasis was placed on termination of pregnancy, here we will illustrate through a multidisciplinary approach and meticulous planning at an expert center, these high-risk women can undergo successful childbirth.

## 1. Introduction

Pulmonary hypertension (PH) comprises a heterogeneous group of diseases that display a unique histological and hemodynamic profile [1] (Table 1). Regardless of etiology, the disease is characterized by elevations in pulmonary pressures which lead to a constellation of abnormal cellular, prothrombotic and vascular constriction effects [2]. These maladaptive changes result in right ventricular (RV) strain and eventually RV failure and death [3]. Pulmonary arterial hypertension (PAH) is a female predominant disease, with early registry data establishing a median age of 36 years [4]. This highlights the clinical relevance to the cardiology and obstetric practice. PAH in pregnancy carries a high maternal and fetal morbidity and mortality, and most modern era guidelines include counseling to avoid pregnancy and even recommend early termination [5]. Despite improved insight and the development of novel targeted therapies, the risk remains unacceptably high; thus, the complex management from pre-conception to post-partum should be undertaken in an experienced center through a multidisciplinary approach.

## 2. Pulmonary Hypertension Overview

The WHO classification system for PH serves the purpose of classifying the pulmonary hypertension population on the basis of unifying pathophysiology, prognosis, and implications for therapy. In 2018, the 6th World Health Symposium refined the five subtypes, using the latest scientific insight to better characterize this disease: pulmonary arterial hypertension (PAH group 1), PH resulting from left heart disease (group 2), PH due to chronic lung disease and/or hypoxia (group 3), PH due to pulmonary artery obstructions (group 4), and PH due to unclear/multifactorial disease (group 5) [6] (Table 1).

Based on data from the US-based REVEAL registry, the leading cause of PAH is idiopathic (40–50% of the cases). Associated PAH from scleroderma, lupus, HIV, portal hypertension, and schistosomiasis are the bulk of the remaining cases. Despite the inciting pandemic of appetite suppressant-induced PAH, medical and illicit drug use account for a minority of cases [7].

The hallmark of pulmonary arterial hypertension is dyspnea. Initially, this complaint may only be present with exercise, but can soon escalate to dyspnea with mild exertion. This can be subtle during pregnancy because this is a normal complaint in pregnancy due to weight gain and increased blood volume in the late 2nd trimester and early 3rd trimester. Once the right ventricle begins to fail, lower extremity edema, abdominal distension, anorexia, and early satiety may be the predominant symptoms. In later stages, syncope and angina may be present and are harbingers for advanced right ventricular failure and low output. Initial evaluation includes an echocardiogram, which is highly sensitive for the detection of pulmonary hypertension and additionally offers insight into the mechanism [8]. If the left heart alone does not explain elevations in pulmonary artery pressure, further testing is pursued to sequentially “rule out” the remaining WHO subgroups. This testing includes pulmonary function testing (PFTs), high-resolution chest computed tomography, overnight oximetry testing for sleep-disordered breathing, and ventilation-perfusion scans. Laboratory diagnostics include autoimmune serologies, HIV serology, and thyroid function. If suspicion remains for Group 1 PAH after all non-invasive evaluation is completed, right heart catheterization remains the gold standard for diagnosis. Right heart catheterization can be done safely in pregnant patients with the use of external shielding, a radial artery approach to avoid pelvic radiation and confining the radiation beam to a smaller area of focus [9]. The hemodynamic profile plays a central role in confirming the presence or absence of PAH, which is defined as a mean pulmonary arterial (mPA) pressure over 20 mm Hg, with a pulmonary capillary wedge pressure (PCWP) under 15 and a pulmonary vascular resistance (PVR) over 3 [1].

## 3. PAH Drug Therapy

Drug therapy has evolved dramatically over the past few decades. During the time of the initial registry studies, there were no approved therapies, and patients were managed with the combination of diuretics, digoxin, and coumadin, based on initial histological findings of the thrombotic phenomenon in the lungs, and improved survival in a retrospective study done in the 1980s [2]. Digoxin has been shown to improve right ventricular contractility and cardiac output in hemodynamic studies; however, there is a paucity of data showing benefit in PAH [10].

The last 3 decades have given rise to several PAH-specific therapies. To date, there are 14 different pharmacological therapies available, all targeting four distinct known mechanisms of vasoconstriction and vasodilation—calcium channels, nitric oxide/cyclic guanosine monophosphate (cGMP), endothelin, and the prostacyclin pathway.

Calcium channel blockade first emerged as a therapeutic option based on a study that showed that a small minority of PAH patients were “responders”, meaning that high-dose calcium channel blockade resulted in a meaningful drop in PVR and mean PA pressures. This is the case in approximately 5% of the PAH population. Given the impracticality of screening with high-dose calcium channel blockers during a hemodynamic right heart catheterization, nitric oxide, adenosine or IV epoprostenol is often used as a rapid screening tool. A decrease in the mean PA by 10 mmHg to a level under 40, without a decrease in cardiac output, denotes a “positive” response—and this population can be considered for high-dose calcium blocker therapy [11].

The nitric oxide/cGMP pathways are a key step in pulmonary vasodilation, and deficiency of this system can lead to adverse vascular remodeling in the pulmonary bed. Phosphodiesterase-5 inhibitors (PDE5i) increase levels of cGMP, therefore leading to downstream vasodilation. Sildenafil and tadalafil are the two PDE5i approved for PAH. They are backed by data showing improvement in the 6 min walk test (sildenafil and tadalafil) as well as time to clinical worsening (tadalafil only). Riociguat is the only Guanylyl cyclase stimulator approved, and it targets directly promoting cGMP production. In addition to improving the above parameters, there is also an improvement in hemodynamics and exercise capacity [12,13].

Endothelin is one of the most potent vasoconstrictors of the pulmonary bed. These levels are increased in pulmonary hypertension patients. Naturally, blocking these receptors would lead to less vasoconstriction. Endothelin receptor antagonist use is backed by improved PVR, increased 6 min walk test, improved functional class, and delayed time to clinical worsening [14].

Finally, the prostacyclin class causes marked pulmonary vasodilation and also carries a strong antithrombotic and anti-inflammatory effect. These are available in oral, inhaled, and parenteral formulations. The important distinction is that the parenteral class of medications are the only FDA approved therapy with strong survival data associated with their use [15]. Of course, this is taken into consideration with the risk associated with continuous intravenous or subcutaneous therapy (infections, abrupt cessation can lead to rebound pulmonary hypertension); therefore, this modality is generally reserved for advanced disease.

With these options, we have seen a marked improvement in outcomes of the pulmonary arterial hypertension population. The pregnant patient poses a unique challenge, as some of these therapies are inherently teratogenic, and these points will be highlighted in the proceeding discussion.

## 4. Physiologic Changes in a Normal Pregnancy

Pregnancy causes several hormonal and mechanical alterations that affect nearly every organ system in order to promote healthy fetal development. Some of these adaptive changes can adversely affect the RV and pulmonary vasculature [8]. Broadly, we can characterize the pregnancy effects on the cardiovascular system by volume expansion and decreased afterload.

Plasma volume increases by 40–50% during the third trimester, but red cell mass only increases about 25% above pre-pregnancy levels [6]. This leads to physiological anemia in the pregnant woman. This is favorable for the feto-placental unit because the decreased blood viscosity allows for better perfusion [6]. With a view on the myocardial effects of this volume change, there is an increase in cardiac output driven by both an increase in stroke volume and heart rate in pregnancy. The downstream effect on a “sick” RV may be worsening ischemia due to the inability to accommodate this extra cardiac output demand.

Primarily driven by estrogen and progesterone, vasodilation during pregnancy upregulates the renin-angiotensin system (RAAS). RAAS upregulation leads to more sodium retention, which has harmful implications in a right heart failure patient [16]. Finally, it is important to note the systemic and pulmonary vasodilatory effects in pregnancy. In a normal woman, the increased cardiac output and marked vasodilation will manifest as lower blood pressure, most evident in early pregnancy. In a patient with pulmonary hypertension, the normal reflex vasodilation occurring in the pulmonary bed may be attenuated or absent, thus the higher cardiac output of pregnancy may cause a further rise in pulmonary pressures due to this inability to vasodilate [17]. The combination of higher cardiac output, lower systemic vascular resistance, and higher pulmonary vascular resistance can potentiate RV decompensation.

Several hematological changes during pregnancy can lead to a hypercoagulable state. There is an increase in fibrinogen (pro-coagulant), and a decrease in protein C and S (which are natural anticoagulants). PAH is histologically characterized by the presence of microvascular thrombotic lesions; therefore, the hypercoagulability of pregnancy has the potential to exacerbate this phenomenon [6].

## 5. Approach to the Pregnant Patient

Patients with PAH during pregnancy usually present with pedal edema, shortness of breath, and dyspnea, which all may be evident during a normal pregnancy. Therefore, it can be challenging to differentiate the normal physiologic pregnancy response versus symptoms that may seem out of proportion and indicative of PAH. A low threshold should be placed for an echocardiogram, which is a low-risk and highly sensitive screening measure. Particular attention must be paid to the at-risk group of women, namely those with mixed connective tissue disease, HIV, or known congenital heart disease [18]. Admittedly, there is sparse literature about exercise studies in the pregnant woman as a tool to distinguish normal pregnancy versus cardiopulmonary pathology [19]. For newly diagnosed cases, right heart catheterization with minimal to no radiation is the gold standard and can be done safely during pregnancy [9,20].

Once the diagnosis of PAH is established, it is still recommended that patients should be individually counseled and offered therapeutic abortion in high-risk cases. These women should promptly be referred to a PH experienced center [21]. If the decision is to continue with the pregnancy, serial risk assessment is a cornerstone of management. There has been a significant progression in risk assessment tools for PH since the 1990s NIH-derived risk tool for patients with idiopathic PH was published [22]. As time progressed with novel pulmonary vasodilator-specific therapy, new European risk assessment tools were established, such as the French Pulmonary Hypertension Network (FPHN), the mostly German COMPERA registry (Comparative, Prospective Registry of Newly Initiated Therapies for Pulmonary Hypertension), and SPAHR (Swedish PAH Registry) [23]. These were the first risk assessment tools that used a set of collective variables to risk stratify patients into low-, intermediate-, or high-risk categories. This would later mirror the European Society of Cardiology (ESC)/European Respiratory Society (ERS) multi-variable approach in 2015 [24,25] (Table 2). While this was an important development in establishing a structured approach to these highly complex patients, there were certain limitations. These risk scores applied exclusively to idiopathic, heritable or drug-induced PAH, excluding the other heterogenous PAH groups. In addition, each variable taken into consideration was weighted equally, with one fixed value for each parameter. The Registry to Evaluate Early and Long-Term PAH Disease Management (REVEAL) was then built to refine this concept [25] (Table 3). The recently updated REVEAL 2.0 utilized weighted grades for each variable, as well as “nonmodifiable” vs. “modifiable” variables. Non-modifiable variables, for example, included “WHO group I subgroup” and “Demographics,” while modifiable variables included “vital signs” and “6MWT.” Furthermore, the CARPREG investigators and the WHO Classification of Maternal Cardiovascular Risk sought to derive a risk stratification system for pregnant women with all forms of cardiac disease [26] (Table 4). Despite the evolvement of risk stratification scoring, there exists no one single variable that can precisely capture risk in the heterogeneous PAH patient, especially in the pregnant patient; thus, the combination of variables known to hold prognostic data has broad clinical applications. Irrespective of which risk score is utilized, appropriate evaluation is mandated for timely escalation of therapy and planning of delivery.

## 6. Delivery Considerations

Establishing an expert multidisciplinary team consisting of pulmonary hypertension specialists, obstetricians, critical care specialists, anesthesiologist, and neonatologists is crucial to ensure favorable outcomes for mother and baby. Two classes of medications commonly used to treat PAH, the endothelin receptor antagonist (ERA) and Guanylyl Cyclase stimulator (sGC), are contraindicated in pregnancy [14]. These therapies should ideally be discontinued pre-conception.

If deemed high risk based on established echocardiogram, functional and clinical parameters, parenteral therapy should be considered. In lower-risk patients, combination therapy with oral phosphodiesterase inhibitors and inhaled prostacyclin analogues can be considered. The greatest hemodynamic stress occurs during weeks 20–24 due to the increase in cardiac output and significant plasma expansion [5]. Continuous clinical evaluation of RV function in PAH patients during pregnancy is vital to determine the need for the augmentation of medical therapy.

Labor, delivery, and the immediate post-partum phase of pregnancy also pose great hemodynamic stress to the RV. The physiological response to pain (Valsalva maneuver, hypoxia, acidosis) can adversely affect pulmonary vascular tone and RV function [8]. In most cases, admission to the intensive care unit prior to scheduled delivery is required for medication and fluid status optimization. Swan–Ganz catheterization is not routinely recommended, but may be considered on a case-by-case basis. Any hemodynamically relevant atrial or ventricular arrhythmias should be aggressively addressed, as they tend to be poorly tolerated by the dysfunctional RV. Pulmonary vasodilator therapy should be maintained throughout the pregnancy and labor, with a low threshold to escalate to intravenous prostacyclin or inhaled nitric oxide if there is clinical instability [26]. Pre-labor discussions should include the availability of inotropes, pressors, and in high-risk situations, mechanical support such as veno-arterial extracorporeal membranous oxygenation (VA-ECMO).

There is no robust clinical evidence to support either routine vaginal or cesarean delivery in women with pulmonary arterial hypertension. Considerations include obstetric indications for cesarean delivery, such as prior cesarean delivery or preeclampsia with severe features. Expert consensus recommends cesarean delivery, although there are practical considerations as well as risks with each option that must be reviewed on an individual patient basis [27,28]. Practically, spontaneous delivery is not favored due to the possibility of the expert PAH team not being available off-hours, leading to an inexperienced team conducting a high-risk delivery. Another consideration would be the frequent use of the Valsalva maneuver during vaginal delivery, which increases intrathoracic pressures and decreases pre-load, all detrimental to a sensitive RV. A common drug used to promote uterine contractions, oxytocin, should be cautiously used as it can also increase pulmonary vascular resistance. The benefits of a vaginal birth include lower bleeding complications, fewer infections, less thromboembolic risk, and less abrupt hemodynamic changes compared to a cesarean section [29].

The largest benefit of a cesarean delivery is that it will avoid a lengthy labor period, especially for a nulliparous patient. Cesarean sections are usually planned at around 37 weeks, both to avoid spontaneous labor and to allow for proper fetal maturation [17]. Preeclampsia exists in 7% of all pregnant patients and is most common after 37 weeks; therefore, it is reasonable to time delivery in such a way to avoid this complication.

Analgesia is imperative to avoid rapid hemodynamic shifts. General anesthesia is not recommended in these patients due to the known effects of negative inotropy and increasing pulmonary vascular resistance during intubation. Therefore, regional block with epidural anesthesia with slow and incremental loading or combined low dose spinal-epidural anesthesia is recommended in PAH patients due to its regional activity and low risk of hypotension [21]. Spinal anesthesia involves the injection of anesthesia directly into the fluid sac, while epidural anesthesia is placed just around this sac in the epidural space. Placing anesthetic directly into the fluid sac is often unpredictable and can reach several dermatomes that can cause a rapid rise in block height, leading to peripheral vasodilation, reduction in venous return, and cardiac pre-load, which can be detrimental to the RV during labor and delivery [30].

Parturition and the first post-partum week have been shown to be a vulnerable period for the PAH patient. Blood volume is increased in the hours and days after childbirth due to autotransfusion of blood from the contracting uterus and shifting of peripheral edema from the extravascular compartment into the systemic vasculature [30]. While this is physiologically tolerable in healthy patients, patients with PAH may not be able to accommodate these ongoing shifts that may persist until 24 weeks postpartum. Immediately post-partum, high-risk patients should be observed in the intensive care unit. This is also an opportunity to discuss the implications of repeat pregnancy and options for contraceptives. Progesterone-only methods can be employed in the immediate post-partum period. Long-acting reversible contraception is highly recommended, such as an intrauterine device (both hormonal and non-hormonal) and the progesterone implant. Estrogen should be avoided given the prothrombotic effects, which can be potentiated in the post-partum state [27].

## 7. Our Institutional Experience

In 2015, our institution developed a multidisciplinary approach to these high-risk patients. As shown in Table 5, we highlight our experience with a summary of three consecutive cases seen over the last year. These cases illustrate successful intermediate-risk deliveries of three women with pulmonary arterial hypertension secondary to multiple etiologies. There are many nuances to take into consideration, and we hope to elucidate the complex decision-making process seen in one representative sample.

This is a 22-year-old G1P0 female with a past medical history of Von Willebrand disease, who was referred for an echocardiogram by her obstetrician after a chest X-ray performed during an emergency room visit for an asthma exacerbation was noted to have cardiomegaly. Despite a relative lack of symptoms, she was found to have severe pulmonary hypertension (RVSP of 67.3 mmHg) with preserved right and left ventricular function at 30 weeks gestation.

**Figure 1:**
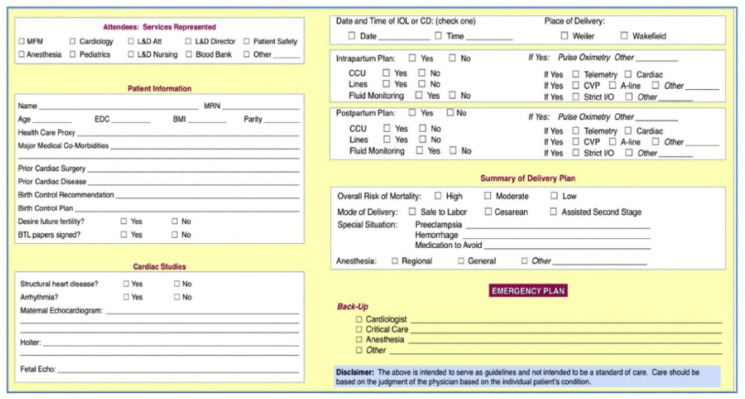
IDT MFM-Cardiology Checklist For Delivery Planning.

The patient was then referred to the MFM-Cardiology Joint Program for further multidisciplinary care. At 32 weeks and 5 days, the patient was scheduled for a right heart catheterization, which was able to be done without the use of fluoroscopy. Her mean pulmonary pressure was found to be 45 mm Hg. In addition, she had low RA and wedge pressures, with an elevated PVR of 4.1 Wood units, consistent with a pre-capillary phenotype. Her autoimmune and HIV testing were negative. Routine PFTs and V/Q scan were deferred until the post-partum period. She was closely followed by echocardiography for the next few weeks. After several multidisciplinary discussions, a decision was made to electively admit the patient at 34 weeks gestation for optimization of PH therapy and planned cesarean section delivery. As shown in Figure 1, an interdisciplinary checklist is completed for delivery planning including date and time of delivery, intra- and postpartum plan, overall mortality risk, and a contingency plan if needed.

During the pre-partum admission, a repeat TTE was done, which showed severe pulmonary hypertension (RVSP of 81.2 mmHg) with moderate right ventricular dilatation and mild to moderate right ventricular hypokinesis. Epoprostenol infusion was initiated and uptitrated to maximally tolerated doses based on symptoms and blood pressure. Cardiothoracic surgery was consulted to ensure ECMO availability and health care proxy forms were in place for the mother and baby. Two days before delivery at 36 weeks gestation, the patient underwent a second right heart catheterization with minimal fluoroscopy for further invasive hemodynamic monitoring. During this catheterization, the patient was placed in a lateral tilt to provide perfusion to the fetus and minimized fluoroscopy time with an abdominal shield to minimize radiation. IV epoprostenol at 27 mg/kg/min was continued during the RHC showing, an improved mean PA pressure of 31 mm Hg, PVR of 3.2 Woods units, and a low RA pressure. The patient was then taken to the OR at 36 weeks gestation for a cesarean delivery due to the development of HELLP syndrome. The PH cardiology team, cardiac anesthesia, high-risk MFM, and NICU teams were all present in the OR. She underwent general anesthesia with slow induction. Although this is not the preferred method, there was a concern for epidural anesthesia in the setting of known Von Willebrand disease, IV prostacyclin (which also intensely inhibits platelets), and abnormal ristocetin assay. She successfully delivered a healthy baby without any peri-operative complications. Over the next 3 days in the cardiac care unit, IV epoprostenol was transitioned to oral therapy after discussions with the patient to forego breastfeeding. An intrauterine device was placed prior to discharge as her preferred birth control method.

## 8. Conclusions and Future Directions

While there have been many pharmacological advances made in the field of pulmonary arterial hypertension, it still poses an extreme threat to the pregnant patient. In the prior era, heavy emphasis was placed on the avoidance or termination of pregnancy. Through a multidisciplinary approach and meticulous planning, these high-risk women can undergo successful childbirth, but this mandates referral to an expert center. There are a multitude of physiological changes that occur in a normal pregnancy that can adversely impact a dysfunctional right ventricle—and awareness and management of these complexities can facilitate a successful outcome.

## Figures and Tables

**Table 1:** Updated WHO Classification From the 6th World Health Symposium in 2018.

1. PAH
1.1. Idiopathic
1.2. Heritable PAH
1.3. Drug and toxin-induced PAH
1.4. PAH associated with:
1.4.1. Connective tissue disease
1.4.2. HIV infection
1.4.3. Portal hypertension
1.4.4. Congenital heart disease
1.4.5. Schistosomiasis
1.5. PAH long-term responders to calcium
channel blockers
1.6. PAH with overt features of venous/
capillaries (PVOD/PCH) involvement
1.7. Persistent PH of the newborn syndrome
2. PH because of left heart disease
2.1. PH because of heart failure with preserved
LVEF
2.2. PH because of heart failure with reduced
LVEF
2.3. Valvular heart disease
2.4. Congenital/acquired cardiovascular
conditions leading to postcapillary PH
3. PH because of lung diseases and/or hypoxia
3.1. Obstructive lung disease
3.2. Restrictive lung disease
3.3. Other lung disease with mixed restrictive/
obstructive pattern
3.4. Hypoxia without lung disease
3.5. Developmental lung disorders
4. PH because of pulmonary artery obstructions
4.1. Chronic thromboembolic PH
4.2. Other pulmonary artery obstructions
5. PH with unclear and/or multifactorial
mechanisms
5.1. Hematological disorders
5.2. Systemic and metabolic disorders
5.3. Others
5.4. Complex congenital heart disease

PAH indicates pulmonary arterial hypertension; PH, pulmonary hypertension.

**Table 2:** ESC/ERS Risk Assessment Guidelines For Pulmonary Arterial Hypertension.

	Low Risk < 5%	Intermediate Risk 5–10%	High Risk > 10%
CLINICAL SIGNS OF RIGHT HEART FAILURE	ABSENT	ABSENT	PRESENT
PROGRESSION OF SYMPTOMS	NO	SLOW	RAPID
SYNCOPE	NO	OCCASIONAL SYNCOPE	REPEATED SYNCOPE
WHO FUNCTIONAL CLASS	I, II	III	IV
6MWD	>440 m	165–440 m	<165 m
CARDIOPULMONARY EXERCISE TESTING	Peak VO2 > 15 mL/min/kg (>65% pred.) VE/VCO2 slope < 36	Peak VO2 11–15 mL/min/kg (35–65% pred.) VE/VCO2 slope 36–44.9	Peak VO2 < 11 mL/min/kg (<35% pred.) VE/VCO2 > 45
NT-proBNP PLASMA LEVELS	BNP < 50 ng/L NT-proBNP < 300 ng/mL	BNP 50–300 ng/L NT-proBNP 300–1400 ng/L	BNP > 300 ng/L NT-proBNP > 1400 ng/L
IMAGING (ECHOCARDIOGRAPHY, CMR IMAGING)	RA area < 18 cm^2^ No pericardial effusion	RA area 18–26 cm^2^ No or minimal, pericardial effusion	RA area > 26 cm^2^ Pericardial effusion
HEMODYNAMICS	RAP < 8 mmHg CI > 2.5 L/min/m^2^ SvO2 > 65%	RAP 8–14 mmHg CI 2.0–2.4 L/min/m^2^ SvO2 60–65%	RAP > 14 mmHg CI < 2.0 L/min/m^2^ SvO2 < 60%

World Health Organization (WHO), 6 min walking distance (6MWD), ventilatory equivalents for carbon dioxide (VE/VCO2), oxygen consumption (VO2), brain natriuretic peptide (BNP), N-terminal pro-brain natriuretic peptide (NT-proBNP), right atrial (RA), Right Atrial Pressure (RAP), Cardiac Index (CI).

**Table 3:** REVEAL Pulmonary Arterial Hypertension Risk Score.

WHO Group I Subgroup	CTD-PAH +1	PoPH +3			Heritable +2
**Demographics **	**Male age > 60 y** **+2**				
**Comorbidities **	**eGFR < 60 mL/min/1.73 m^2^ or renal insufficiency ** **+1**				
**NYHA/WHO Functional Class**	I **−1**	**III** **+1**			**IV** **+2**
**Vital Signs**	**SBP < 110 mm Hg** +1			**HR > 96 BPM** +1	
**All-cause Hospitalizations < 6 mo**	**+1**				
**6MWT**	**>440m** −2	**320 to <440 m** **−1**			**<165 m** **+1**
**BNP**	**<50 pg/mL or NT-proBNP < 300 pg/mL** −2	**200 to <800 pg/mL ** **+1**			**>800 pg/mL or NT-proBNP > 1100 pg/mL** **+2**
**Echocardiogram**	**Pericardial effusion** +1				
**Pulmonary Function test**	**% Predicted DLCO < 40%** +1				
**Right Heart Catheterization**	**mRAP > 20 mm Hg within 1 y** +1			**PVR < 5 Woods units** −1	
Sum Of Above is Risk Score:					
Low Risk			Score ≤ 6		
Intermediate Risk			Score 7–8		
High Risk			Score ≥ 9		

PAH associated with connective tissue disease (CTD-PAH), Diffusing capacity of the lungs for carbon monoxide (DLCO), estimated glomerular filtration rate (eGFR), Functional category (FC), heart rate (HR), mean right atrial pressure (mRAP), New York Heart Association (NYHA), Pulmonary arterial hypertension (PAH), Pulmonary arterial hypertension associated with portopulmonary hypertension (PoPH), Pulmonary vascular resistance (PVR), Registry to Evaluate Early and Long-Term Pulmonary Arterial Hypertension Disease Management (REVEAL), Systolic BP (SBP).

**Table 4:** WHO Classification of Maternal Cardiovascular Risk.

**WHO I:**
Uncomplicated, small or mild:
Pulmonary stenosis
Patent ductus arteriosus
Mitral valve prolapse
Successfully repaired simple shunt defects (ASD, VSD, PDA, APVR)
Delivery: low risk, delivery at local hospital
**WHO II:**
Unoperated atrial or ventricular septal defect
Repaired TOF
Most arrhythmias
Delivery: small to moderate risk, delivery at local hospital
**WHO II-III:**
Mild LV impairment
Hypertrophic cardiomyopathy
Native or tissue valvular disease not considered WHO I or IV
Marfan syndrome w/o aortic dilatation
Aorta < 45 mm in aortic disease associated with bicuspid aortic valve
Repaired coarctation
Delivery: intermediate to high risk, delivery at expert center
**WHO III:**
Mechanical valve
Systemic RV with good or mild impairment
Fontan circulation
Unrepaired cyanotic heart disease
Other complex congentital heart disease
Aortic dilatation 40-45 mm in Marfan syndrome
Aortic dilatation 45-50 mm in aortic disease associated with bicuspid aortic valve
Delivery: high risk, delivery at expert center
**WHO IV: Pregnancy contraindicated**
PAH of any case
Severe systemic ventricular dysfunction (LVEF < 30%, NYHA III-IV)
Previous peripartum cardiomyopathy with any residual impairment of LV function
Severe MS, severe symptomatic AS
Marfan syndrome with aorta dilated > 45 mm
Aortic dilatation > 50 mm in aortic disease associated with bicuspid aortic valve
Native severe coarctation
Delivery: very high risk, delivery at expert center

Atrial sepal defect (ASD), ventricular septal defect (VSD), Patent ductus arteriosus (PDA), Anomalous pulmonary venous drainange (APVR), Tetralogy of Fallot (TOF), Mitral stenosis (MS), Aortic Stenosis (AS).

**Table 5:** PAH and pregnancy management in three representative cases.

Case	Age at Delivery (Years Old)	WHO Group 1 Etiology	PAH Therapy	PVR (Invasive Woods, Units)	Mean Pulmonary Arterial Pressure (mmHg)	Risk Score	Mode of Delivery	Outcome
1	19	SLE	IV Sildenafil, Prostacyclin	15	50	Intermediate	C-section	Healthy Maternal and Fetal Status
2	22	Idiopathic	IV Sildenafil, Prostacyclin	4.1	45	Intermediate	C-section	Healthy Maternal and Fetal Status
3	30	SLE	Sildenafil	6.9	42	Intermediate	Vaginal	Healthy Maternal and Fetal Status
